# Psychosocial health risks among nursing staff working in shifts

**DOI:** 10.3389/fpubh.2026.1749277

**Published:** 2026-04-13

**Authors:** Kamila Rachubińska, Ewa Kupcewicz, Anna Maria Cybulska, Hanna Stępień, Elżbieta Grochans, Daria Schneider-Matyka

**Affiliations:** 1 Department of Nursing, Pomeranian Medical University in Szczecin, Szczecin, Poland; 2 Department of Nursing, Collegium Medicum, University of Warmia and Mazury in Olsztyn, Olsztyn, Poland; 3 Student Research Club at Department of Nursing, Faculty of Health Sciences, Pomeranian Medical University, Szczecin, Poland

**Keywords:** burnout, fatigue, insomnia, nurses, shift work schedule, stress disorders

## Abstract

**Background:**

Nurses are one of the professional groups most exposed to psychosocial risks. Excessive responsibilities, time pressure, the need to make quick decisions, shift work, lack of adequate rest, contact with death and human suffering – all these factors can significantly affect the mental state and result in the development of health problems among nursing staff.

**Materials and methods:**

The study was conducted among 193 nurses working at the University Clinical Hospital No. 2 of the Pomeranian Medical University in Szczecin. Participation in the study was anonymous and voluntary. The study used a proprietary questionnaire and the following standardized tools: PSS-10, FAS, AIS and MBI.

**Objective:**

The aim of the study was to identify psychosocial health risks among nursing staff working in a shift system.

**Results:**

More than half of the respondents (52.85%) showed moderate fatigue according to FAS. It was found that 66.32% of respondents suffered from insomnia according to AIS, 66.84% of respondents experienced high levels of stress according to PSS-10. It was shown that 45.60% of respondents had high levels of emotional exhaustion, 43.01% had high levels of depersonalization, and as many as 73.06% had high levels of lack of accomplishment according to MBI. Fatigue (FAS) and insomnia (AIS) showed significant positive correlations with emotional exhaustion and depersonalization, as well as negative correlations with a sense of professional achievement.

**Conclusion:**

Overall, the findings indicate that shift work constitutes a significant psychological health risk for nurses, with stress, fatigue, sleep disturbances, and burnout forming a closely interrelated cluster of adverse outcomes. The observed associations suggest that chronic occupational strain may reinforce emotional exhaustion, depersonalization, and reduced professional efficacy. Moreover, work organization and length of service appear to play an important role in shaping nurses’ vulnerability to these risks. These results highlight the need for targeted organizational and preventive strategies aimed at reducing psychosocial burden and supporting mental well-being among shift-working nursing staff.

## 1 Introduction

Working as a nurse involves not only responsibility for patients’ health and lives but also constant exposure to significant mental and physical demands. Psychosocial risks—defined as aspects of work organization, management, and the social and environmental context that may cause psychological, social, or physical harm (1)—are particularly prevalent in the nursing profession. Previous research has shown that these risks arise both from the content of work (e.g., workload, time pressure, working hours) and from its organizational and social context, including management style, organizational culture, role ambiguity, and interpersonal relationships (1, 2). In nursing practice, these factors often coexist and interact, potentially amplifying their negative impact on mental health.

Although numerous studies have examined selected psychosocial risks in nursing, the existing literature remains fragmented. Most available research focuses on individual outcomes—such as stress, burnout, or sleep disturbances—analyzed separately and often in heterogeneous professional groups. As a result, there is still limited understanding of how key psychological health risks co-occur and interact among nurses working in shift systems, particularly when stress, fatigue, sleep quality, and burnout are examined simultaneously within the same organizational context.

### 1.1 Occupational stress

Occupational stress is one of the most frequently described psychosocial hazards in nursing. Stress responses may range from adaptive and motivating to chronic and destructive, ultimately leading to exhaustion and health deterioration (3–5). Nurses are exposed to multiple stressors, including responsibility for patients’ lives, decision-making under time pressure, and repeated exposure to suffering and death (6–11). While stress among nurses has been extensively documented, less is known about how perceived stress relates to other psychosocial outcomes—such as fatigue, sleep disturbances, and burnout—particularly in shift-working nurses.

### 1.2 Fatigue

Fatigue is a common consequence of prolonged workload and insufficient recovery, especially in shift work systems. It is characterized by mental and physical exhaustion, reduced concentration, and decreased motivation (12). Previous studies have shown that fatigue negatively affects work performance and patient safety (13–15). However, despite evidence that fatigue frequently coexists with stress and sleep disorders, the interrelationships between these phenomena in nursing populations remain insufficiently explored.

### 1.3 Sleep disorders

Shift work, especially night work, disrupts circadian rhythms and is strongly associated with sleep disturbances, including insomnia and excessive daytime sleepiness (16–20). Sleep deprivation has been linked to numerous adverse health outcomes, such as impaired immune function, metabolic disorders, cognitive errors, and increased cardiovascular risk (21). Although sleep problems among nurses are well recognized, their combined impact with stress and fatigue on burnout dimensions has not been consistently analyzed in empirical studies.

### 1.4 Burnout syndrome

Burnout is particularly prevalent in care professions and is conceptualized as a syndrome comprising emotional exhaustion, depersonalization, and reduced personal accomplishment (22, 23). Burnout has been associated with chronic occupational stress, excessive workload, and lack of organizational support (24–27). While burnout among nurses has been widely studied, there is still a lack of integrative research examining burnout in relation to stress, fatigue, and sleep quality within shift-working nursing staff.

### 1.5 Shift work as a multidimensional risk factor

Shift work refers to work schedules that extend beyond the traditional daytime hours and involve rotating, evening, night, or irregular shifts. In healthcare settings, shift work is necessary to ensure continuous patient care; however, it disrupts the natural circadian rhythm and sleep–wake cycle. This disruption has been associated with multiple adverse health outcomes among healthcare workers, including fatigue, sleep disturbances, psychological stress, and burnout. Because these effects often occur simultaneously and influence one another, shift work can be considered a multidimensional occupational risk factor affecting both physical and psychological well-being (28). Previous studies have documented associations between shift work and stress, fatigue, sleep disorders, and burnout. Nevertheless, many of these studies have focused on single outcomes or have not adequately accounted for work-related and sociodemographic factors that may modify these relationships.

Given the multidimensional and interrelated nature of psychosocial risks in nursing, there is a clear need for studies that examine stress, fatigue, sleep quality, and burnout simultaneously, particularly among nurses working in shift systems and within a uniform organizational setting. Such an approach allows for a more comprehensive understanding of psychological health risks and their determinants.

Therefore, the aim of this study was to assess the level of psychosocial health risks among nurses working in a shift system, with particular emphasis on perceived stress, fatigue, sleep quality, and burnout, while considering selected sociodemographic and work-related variables. The findings may contribute to filling existing gaps in the literature and provide evidence to support the development of targeted preventive and organizational interventions aimed at improving nurses’ psychological well-being.

## 2 Methods

### 2.1 Participants

This cross-sectional observational study was carried out at a single point in time among 193 nurses employed at the University Clinical Hospital No. 2 in Szczecin. The research was carried out from October to December 2024.

### 2.2 Sampling technique

A cross-sectional observational design was applied, and participants were recruited using a convenience sampling technique from the University Clinical Hospital No. 2 in Szczecin. Prior to data collection, permission to conduct the study was obtained from each participating institution. The study was conducted in a single healthcare facility to ensure a homogeneous organizational and work-environment context. This design allowed for better control of institutional factors such as staffing models, shift schedules, workload organization, management structure, and internal policies, which are known to influence psychosocial health outcomes among nurses. By limiting variability related to organizational differences, the study aimed to more accurately examine the relationships between stress, fatigue, sleep quality, and burnout within a uniform working environment.

### 2.3 Inclusion and exclusion criteria

The criteria for inclusion in the study were: informed and voluntary consent to participate, employment as a nurse in a ward of USK No. 2 in Szczecin, working on call (shift system including 12-h day and night shifts). The exclusion criteria were: not working on a shift basis, not being licensed to practise as a nurse, and not consenting to participate in the study.

The size of the research sample was determined on the basis of statistical data on the number of nurses with an active licence to practise, working in the city of Szczecin, in the West Pomeranian Province, in 2024. In the year under review, 4,500 people were professionally active. The confidence level was set at 95%, the maximum error at 6%, and the estimated fraction size at 0.5. The total number of professionally active nurses qualified to participate in the study was 252. A total of 250 survey sets were distributed, of which 193 questionnaires were qualified for statistical analysis after verification of data completeness, corresponding to a return rate of 83%.

After obtaining the consent of the hospital management, trained researchers distributed paper versions of the questionnaires to the respondents. The study was anonymous and voluntary, and participants did not receive any remuneration for their participation. Trained interviewers provided participants with detailed information about the purpose of the study, its course and the possibility of asking questions. Each respondent had the right to withdraw from participation at any time without giving a reason. Completing the questionnaire took an average of about 15 min.

### 2.4 Data collection

The study used a diagnostic survey method with a questionnaire technique. Standardised research tools and a proprietary questionnaire were used to collect data.

The questionnaire consisted of 11 questions. The first part concerned socio-demographic data such as age, gender, marital status, place of residence and education. The second part concerned seniority and place of work (conservative ward, surgical ward, maternity ward, intensive care unit, hospital emergency department).

The Perceived Stress Scale (PSS-10), authored by Sheldon Cohen, Tom Kasmarck and Robin Mermelstein, adapted into Polish by Zygfryd Juczyński and Nina Ogińska-Bulik. It consists of 10 questions about subjective feelings related to personal events, problems, behaviour and coping. Respondents rate the frequency of a given emotion over the last month on a 5-point scale. The results are interpreted using standard norms, where a score of 1–4 indicates a low level of stress, 5–6 indicates a medium level of stress, and 7–10 indicates a high level of stress. The internal reliability of the scale, assessed based on Cronbach’s alpha, ranges from 0.84 to 0.86 (29).

Fatigue Assessment Scale (FAS). It consists of 5 questions about physical symptoms and 5 questions about mental symptoms related to fatigue that have occurred over the past year. A minimum of 10 points and a maximum of 50 points can be obtained. The interpretation of the results is as follows: <22 points – no fatigue, 22–34 indicates mild fatigue, and a score above >34 points indicates very severe fatigue. High internal consistency and reliability for each scale were revealed: the Cronbach alpha values were 0.92 and 0.95 for the FSS and FSS, respectively (30).

Athens Insomnia Scale (AIS) – assesses the severity of insomnia symptoms using the ICD-10 diagnostic criteria. It consists of 8 questions about symptoms that have occurred at least 3 times in the last month. The questions concern: falling asleep, waking up at night, sleep quality, total sleep time, well-being after a night’s sleep, daytime sleepiness, and physical and mental performance after a night’s sleep. The interpretation of the results is as follows: <5 points no insomnia (normal), 6–10 points indicates borderline normal, i.e., sleep hygiene rules should be followed, and medical consultation is necessary if the condition worsens, >10 points indicates insomnia. The internal consistency (Cronbach’s alpha = 0.90) and the test–retest reliability (r^2^ = 0.92) of the AIS were found to be very satisfactory (31).

The Maslach Burnout Inventory (MBI) questionnaire was developed by Maslach and Jackson and adapted into Polish by Tomasz Pasikowski. It consists of 22 questions in three subscales: 9 questions related to emotional exhaustion (EE), 5 questions related to depersonalisation (DEP) and 8 questions related to reduced personal accomplishment (PA). Responses were recorded using a frequency-based Likert scale. A score of 0 to 100 can be obtained, with a higher score indicating a higher risk of burnout. An average is also calculated from the three subscales, which indicates the overall burnout index. Cronbach’s alpha values indicated high internal consistency for EE (0.90), DEP (0.89), and PA (0.83) (32).

### 2.5 Data analysis

The analysis of quantitative variables was performed by calculating descriptive statistics such as: mean, standard deviations, median, quartiles, and minimum and maximum. The analysis of qualitative variables was performed by calculating the absolute frequency and percentage occurrence of all values that these variables could take. The comparison of the results of the FAS, AIS, PSS-10 and MBI questionnaires in two groups was performed using the Mann–Whitney test. The comparison of the results of the FAS, AIS, PSS-10 and MBI questionnaires in three or more groups was performed using the Kruskal-Wallis test, and in the event of statistically significant differences between the groups, Dunn’s post-hoc test. Correlations between the results of the FAS, AIS, PSS-10 and MBI questionnaires were analysed using Spearman’s correlation coefficient. A significance level of 0.05 was adopted in the analysis, meaning that all *p*-values below 0.05 were interpreted as indicating significant correlations. The analysis was performed using R software, version 4.3.2 (33).

### 2.6 Ethical considerations

The study was conducted in accordance with the Declaration of Helsinki and the principles of research ethics. A positive opinion was obtained from the Bioethics Committee of the Pomeranian Medical University in Szczecin KB-0012/219/06/16 and the hospital management’s consent to conduct the study.

## 3 Results

### 3.1 Participants

The study involved 193 participants, 75.13% women and 24.87% men, who were nursing staff working at the Independent Public Clinical Hospital No. 2 of the Pomeranian Medical University in Szczecin. The most numerous age group was 41 to 50 years old, and the average age of respondents was 44.87 years. Most of the respondents had a bachelor’s degree in nursing (38.86%) and a master’s degree (28.50%). Married persons constituted the largest group among the respondents – 35.75%. The largest group of respondents lived in cities with a population of 10,000 to 100,000–44.04%.

More than half of the respondents (59.59%) were employed under an employment contract, and 31.09% under a civil law contract. The largest group of respondents were people who had been working in the profession for 21 to 30 years – 30.57% and in conservative wards – 46.11%.

### 3.2 Description of the level of fatigue according to FAS, sleep disorders according to AIS, stress according to PSS-10 and burnout according to MBI among nursing staff

The FAS questionnaire allows the level of fatigue in the respondent to be assessed. More than half of the respondents (52.85%) felt moderate fatigue, 34.72% had no symptoms of fatigue, and 12.44% felt severe fatigue. Based on the results of the Athens Insomnia Scale (AIS), it was found that 66.32% of respondents suffered from insomnia. The PSS-10 questionnaire allows for the assessment of the severity of subjectively perceived stress. It was shown that 66.84% of respondents had high stress levels and 21.24% had medium stress levels. The Maslach Burnout Inventory (MBI) questionnaire allows for the assessment of burnout in three aspects: emotional exhaustion, depersonalisation, and a sense of lack of accomplishment. The study showed that 45.60% of respondents had a high level of emotional exhaustion, and 34.20% had a medium level. It was shown that 43.01% of respondents had a high level of depersonalisation and 35.23% had a medium level of depersonalisation. The study showed that the majority (73.06%) had a high level of feeling of lack of accomplishment and 18.13% had a medium level (Table 1).

**Table 1:** Level of perceived fatigue according to FAS, sleep disorders according to AIS, stress according to PSS-10 and burnout according to MBI.

Name of standarised tool	Number of points	Interpretation	*n*	%
FAS	10–21	No fatigue	67	34.72
	22	Moderate fatigue	102	52.85
	35	Severe fatigue	24	12.44
AIS	0–5	No insomnia	65	33.68
	6	Insomnia	128	66.32
PSS-10	0–13	Low stress level	23	11.92
	14	Medium stress level	41	21.24
	Above 19	High stress level	129	66.84
MBI emotional exhaustion	0–16	Low level	39	20.21
	17	Medium level	66	34.20
	Above 26	High level	88	45.60
MBI depersonalisation	0–6	Low level	42	21.76
	7–12	Medium level	68	35.23
	Above 12	High level	83	43.01
MBI feeling of lack of achievement	0–31	High level	141	73.06
	32	Medium level	35	18.13
	Above 38	Low level	17	8.81

PPS-10 the perceived stress scale, FAS, fatigue assessment scale, AIS, Athens insomnia scale, MBI, Maslach Burnout Inventory.

### 3.3 Effects of sociodemographic variables on fatigue severity according to FAS, sleep disorders according to AIS, stress according to PSS-10 and burnout according to MBI

Data analysis showed a statistically significant, moderate correlation between fatigue according to FAS and age (*p* < 0.001). The correlation is moderate and positive (0.574), meaning that the older the age, the higher the level of fatigue.

**Figure 1:**
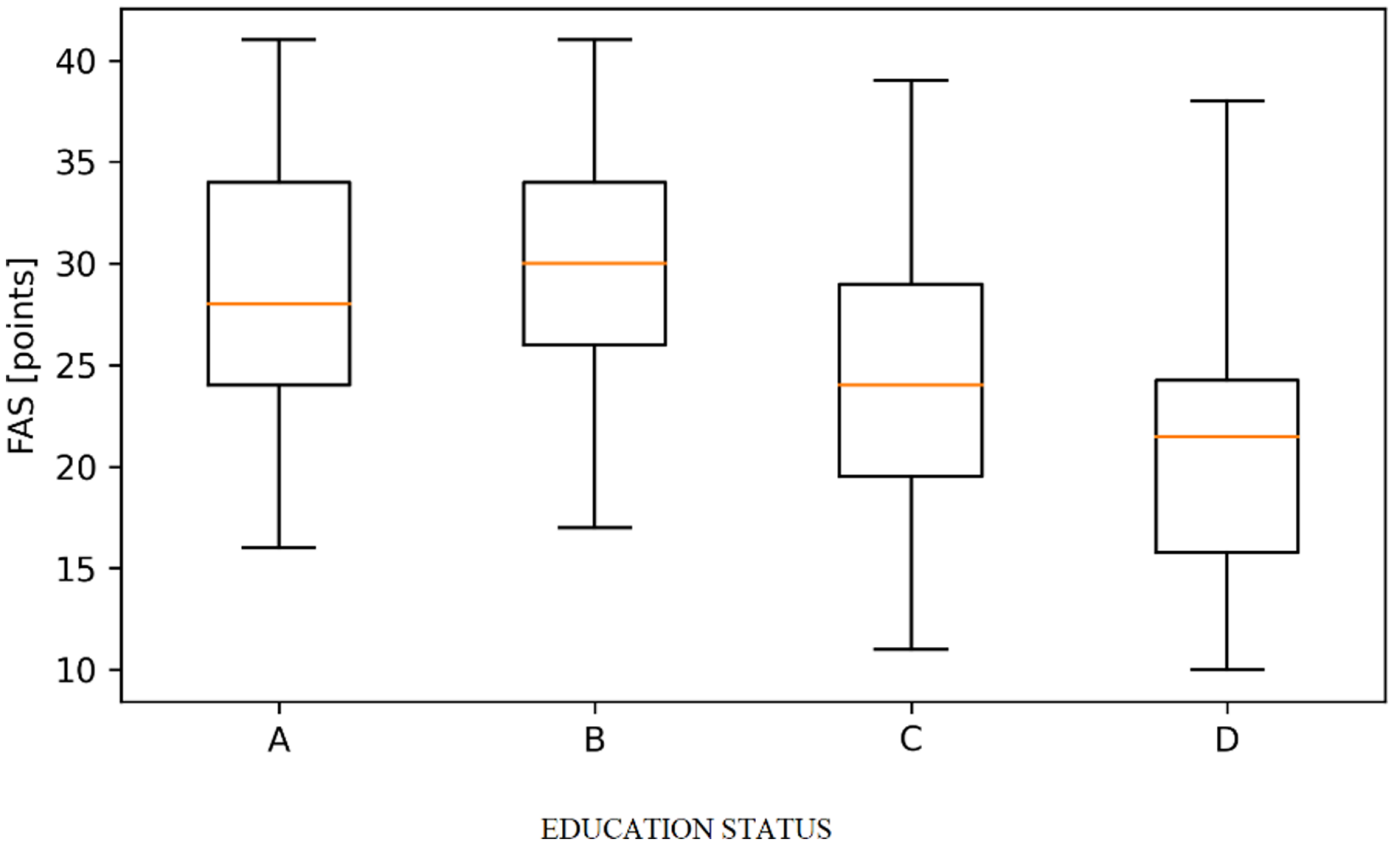
Distribution of fatigue assessment scale (FAS) scores according to education level. (A - Medical High School, B - Vocational studies, C - Bachelor’s degree in nursing, D - Master’s or Doctorate in Nursing). Boxplots represent the median, interquartile range (Q1–Q3), and minimum–maximum values. Statistically significant differences between groups were observed (Kruskal–Wallis test, *p* < 0.001).

**Figure 2:**
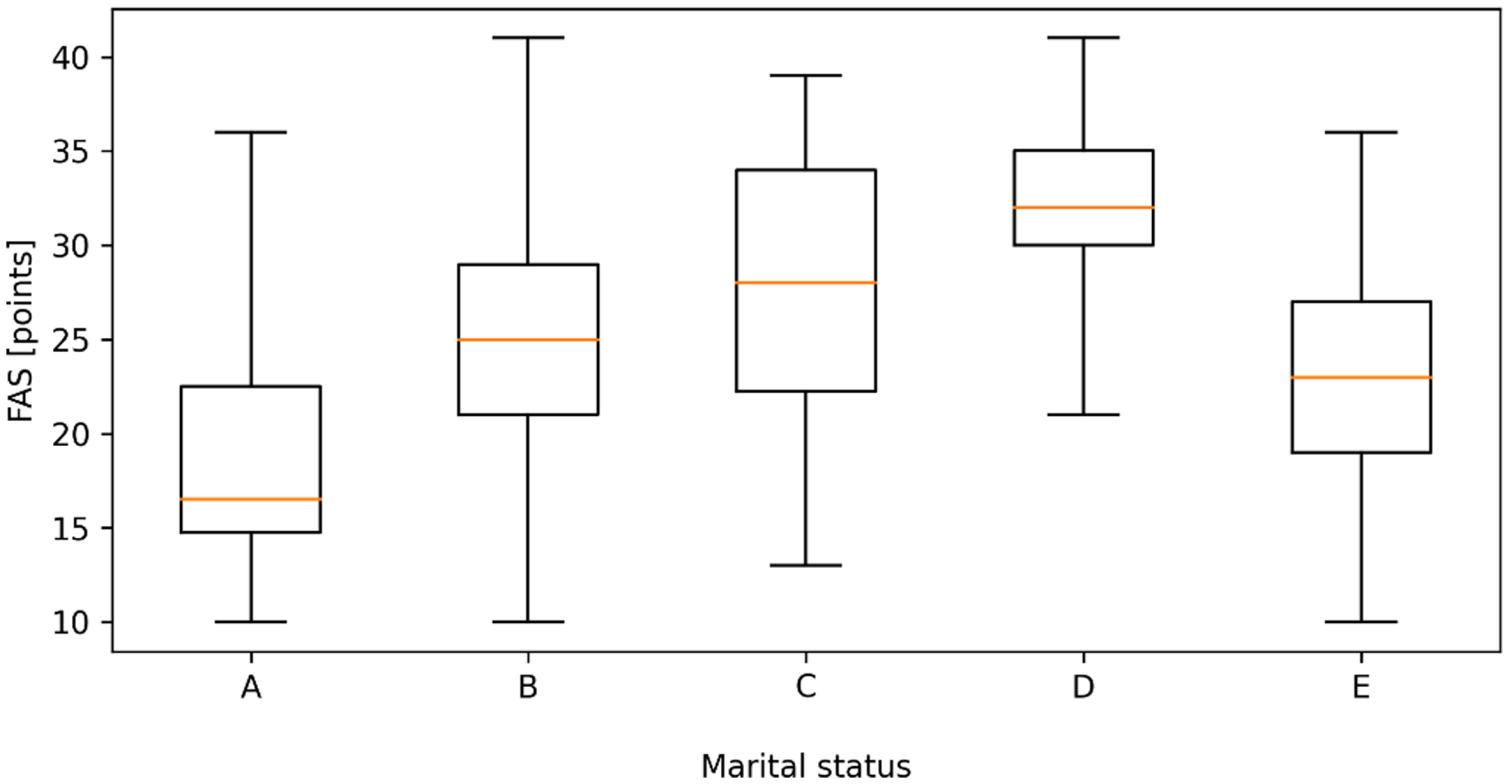
Distribution of fatigue assessment scale (FAS) scores according to marital status. (A - Single, B - Married, C - Divorced, D - Widow/widower, E - In an informal relationship). Boxplots represent the median, interquartile range (Q1–Q3), and minimum–maximum values. Statistically significant differences between groups were observed (Kruskal–Wallis test, *p* < 0.001).

Data analysis showed a statistically significant difference (*p* < 0.05) in fatigue according to FAS depending on the education and marital status of the respondents. The level of fatigue was significantly higher in people with vocational education (M = 29.08) and medical high school education (M = 28.22) than in people with a bachelor’s degree (M = 24.49) and significantly higher in people with a master’s degree and a doctorate (M = 21.09). The level of fatigue was significantly higher: in widowed persons than in married persons (M = 32.15 vs. 24.93), in persons living in informal relationships and in unmarried persons than in divorced persons (22.59; M = 19.15 vs. M = 27.64); (Figures 1, 2).

Data analysis showed a statistically significant positive, moderate correlation (0.565; *p* < 0.001) between the severity of sleep disorders according to AIS and the age of the respondents. The result indicates that the older the age, the more severe the insomnia.

**Figure 3:**
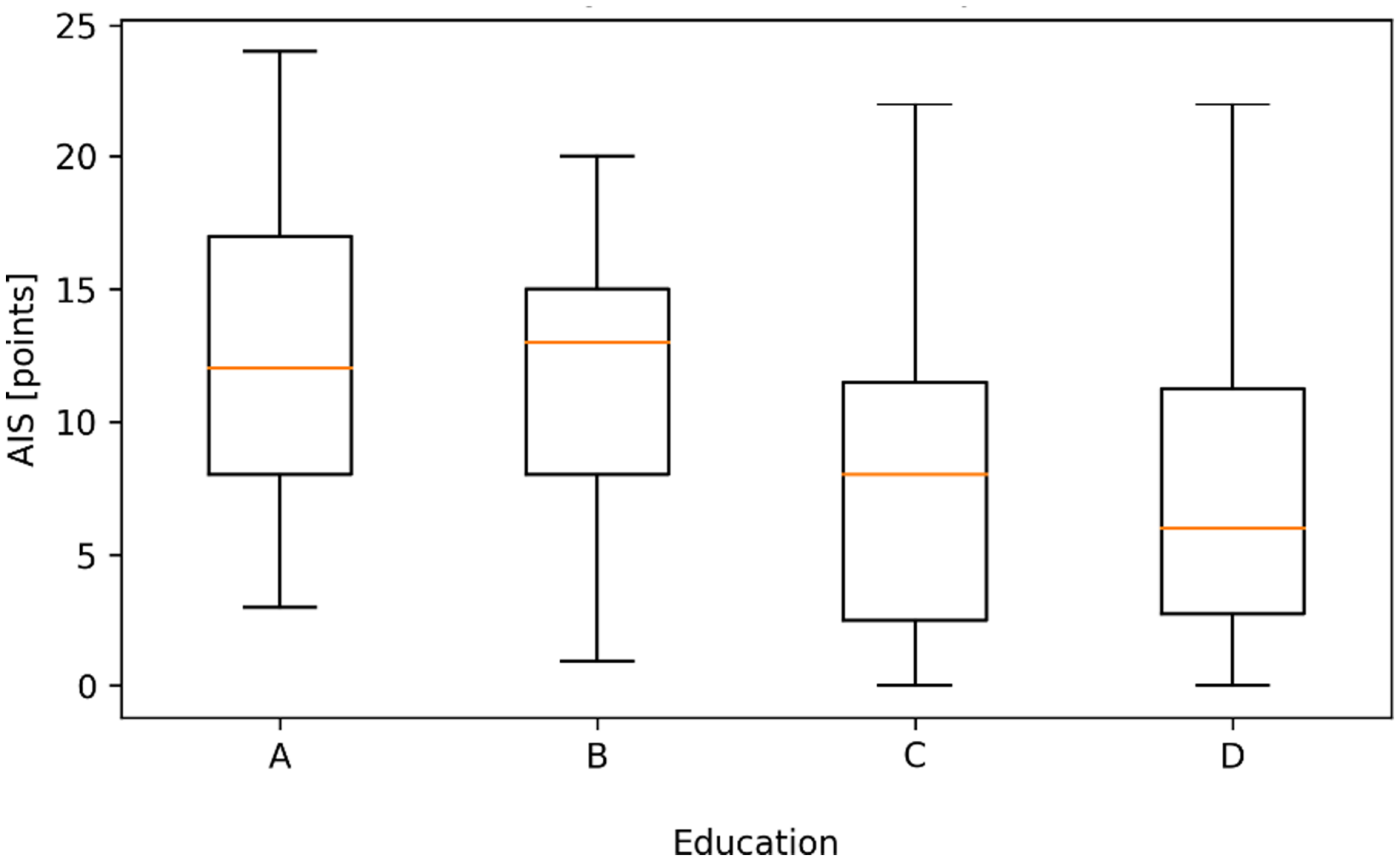
Distribution of Athens insomnia scale (AIS) scores according to education level. (A - Medical high school B - Vocational studies C - Bachelor’s degree in nursing D - Master’s or Doctorate in Nursing). Boxplots represent the median, interquartile range (Q1–Q3), and minimum–maximum values. Statistically significant differences between groups were observed (Kruskal–Wallis test, *p* < 0.001).

**Figure 4:**
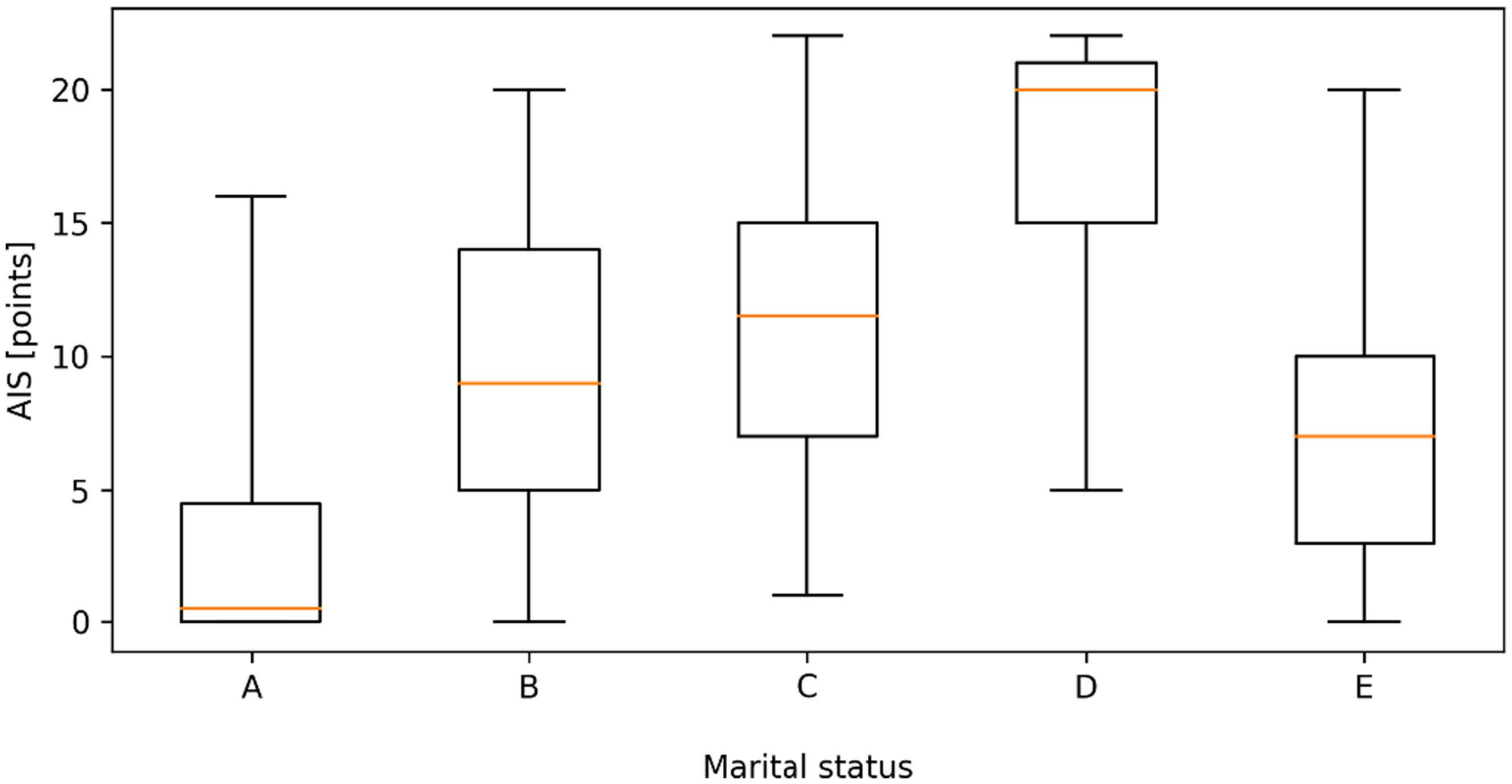
Distribution of Athens insomnia scale (AIS) scores according to marital status. (A - Single, B - Married, C - Divorced, D - Widow/widower, E - In an informal relationship). Boxplots represent the median, interquartile range (Q1–Q3), and minimum–maximum values. Statistically significant differences between groups were observed (Kruskal–Wallis test, *p* < 0.001).

Data analysis showed a statistically significant difference (*p* < 0.05) in sleep disorders according to AIS depending on the education and marital status of the respondents. Insomnia was significantly more severe in people with vocational education and medical high school education than in people with a bachelor’s degree and people with a master’s or doctoral degree (12.43; 11.60 vs. 7.77; 7.32). Insomnia was significantly more severe in widowed individuals than in divorced individuals and married individuals (17.31 vs. 11.10; 9.28), where in turn it was significantly more severe than in people living in informal relationships, where in turn it was significantly more severe than in single people (Figures 3, 4).

Data analysis showed a statistically significant, weak correlation (*p* = 0.006) between stress severity according to PSS-10 and the age of respondents (0.197).

**Figure 5:**
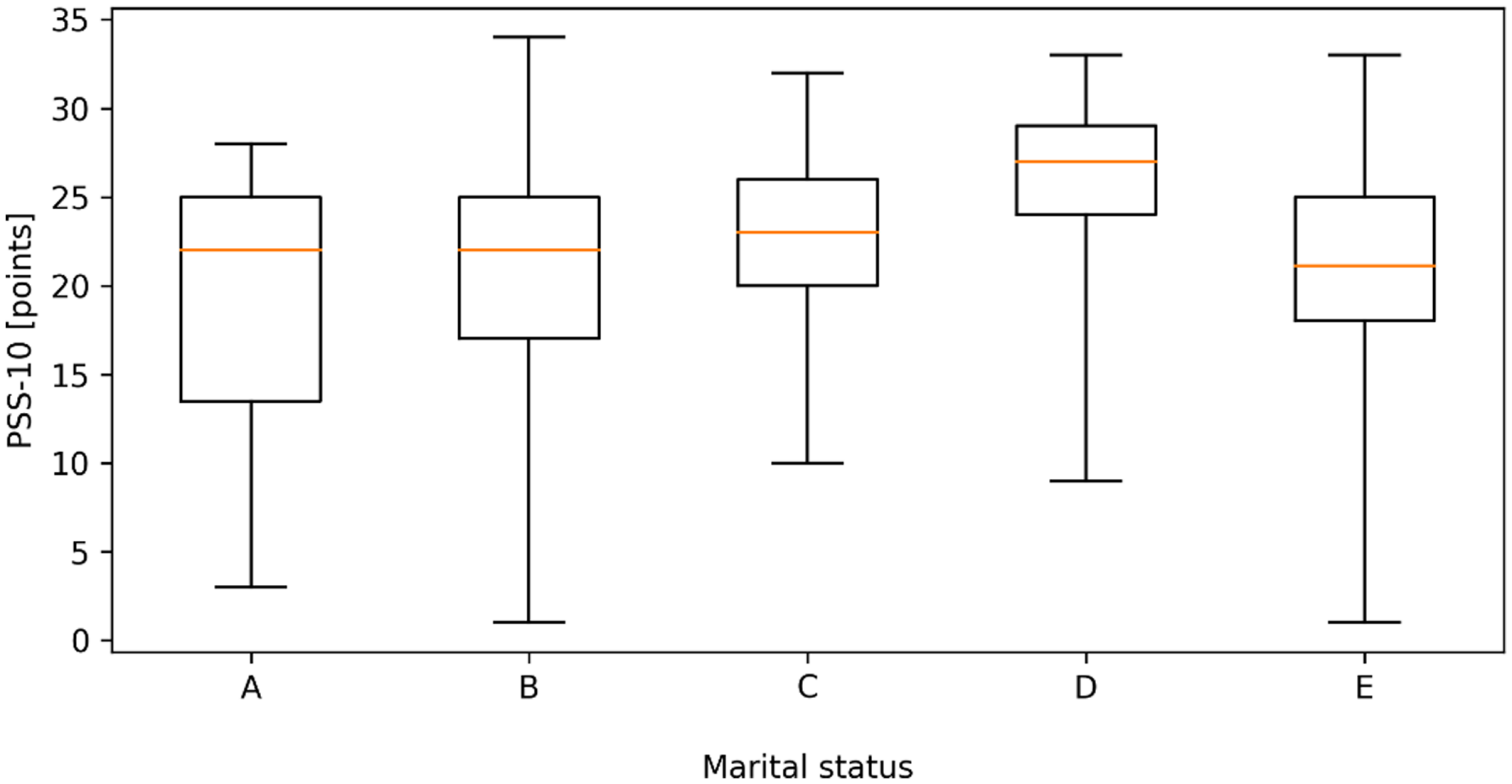
Distribution of perceived stress scale (PSS-10) scores according to marital status (A - Single, B - Married, C - Divorced, D - Widow/widower, E - In an informal relationship). Boxplots represent the median, interquartile range (Q1–Q3), and minimum–maximum values. Statistically significant differences between groups were observed (Kruskal–Wallis test, *p* = 0.015).

No statistically significant differences (*p* > 0.05) were found in the severity of stress according to PSS-10 depending on the education level of the respondents. Data analysis showed a statistically significant difference (*p* < 0.05) in the severity of stress depending on the marital status of the respondents. The level of stress was significantly higher in widowed people than in married people, single people and people living in informal relationships (25.38 vs. 20.75; 18.80; 20.86); (Figure 5).

The age of respondents correlates significantly (*p* < 0.05) and positively (*r* > 0) with emotional exhaustion and depersonalisation according to MBI, i.e., the older the age, the greater the emotional exhaustion and the higher the level of depersonalisation. The age of respondents correlates significantly (*p* < 0.05) and negatively (*r* < 0) with a sense of lack of accomplishment, so the older the age, the greater the sense of lack of accomplishment (lower number of points); (Table 2).

**Table 2:** Relationship between burnout according to the MBI and the age of respondents.

Variable	MBI					
	Emotional exhaustion		Depersonalisation		Sense of lack of accomplishment	
	*r*	*p*	*r*	*p*	*r*	*p*
Age [years]	0.522	<0.001	0.425	<0.001	−0.472	<0.001

MBI, Maslach Burnout Inventory.

Data analysis showed that values *p* < 0.05 indicate statistically significant differences:

Emotional exhaustion and depersonalisation were significantly higher in people with vocational qualifications and medical high school qualifications than in people with bachelor’s degrees and people with master’s or doctoral degrees. The feeling of lack of achievement was significantly lower (higher number of points) in people with a master’s or doctoral degree and in people with a bachelor’s degree than in people with a medical high school education and in people with vocational education. Emotional exhaustion was significantly greater among widowed individuals than among divorced individuals, where in turn it was significantly greater than among individuals living in informal relationships and among married individuals, where in turn it was significantly greater than among single individuals.

**Figure 6:**
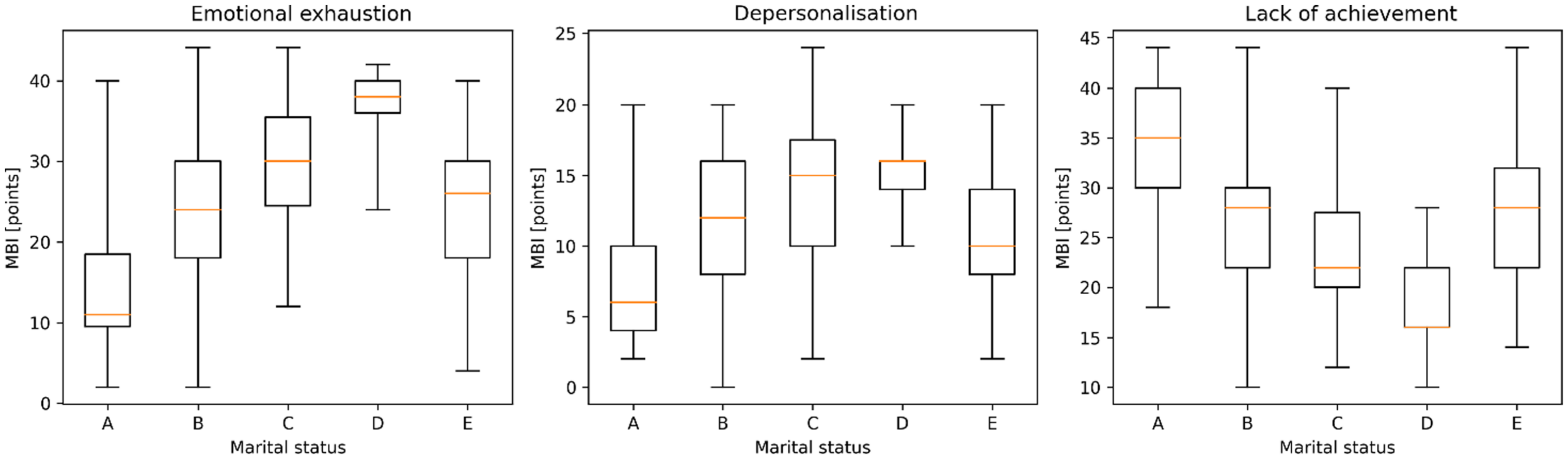
Maslach Burnout Inventory (MBI) subscale scores according to marital status. (A - Single, B - Married, C - Divorced, D - Widow/widower, E - In an informal relationship). Boxplots present the distribution of emotional exhaustion, depersonalization, and reduced personal accomplishment across marital status groups. Boxplots represent the median, interquartile range (Q1–Q3), and minimum–maximum values. Statistically significant differences were observed between groups (Kruskal–Wallis test, *p* < 0.001).

Depersonalisation was significantly more intense among widowed and divorced people than among married people, people living in informal relationships, and single people, and among married people than among single people. The feeling of lack of achievement was significantly lower (higher scores) among single people than among those living in informal relationships and married people, where in turn it was significantly lower (higher scores) than among divorced people, where in turn it was significantly lower (higher scores) than among widowed people (Figure 6).

### 3.4 Fatigue according to FAS, sleep disorders according to AIS, stress according to PSS-10 and burnout according to MBI depending on seniority and place of work

The data analysis did not reveal any statistically significant differences in the severity of fatigue according to FAS depending on the workplace (*p* > 0.05). However, a statistically significant moderate positive correlation (*p* < 0.001) was found between fatigue and the length of service of the respondents, with longer service resulting in higher levels of fatigue (0.534).

The data analysis did not reveal any statistically significant differences (*p* > 0.05) in the severity of sleep disorders according to the AIS depending on the workplace. However, a statistically significant positive, moderate correlation (0.571; *p*<0.001) between sleep disorders according to the AIS and the length of service of the respondents, with longer service in the profession correlating with more severe insomnia in the respondents.

The data analysis did not show any statistically significant differences in the level of stress according to PSS-10 depending on the workplace (*p* > 0.05). A statistically significant, weak correlation (0.195; *p* = 0.007) was found between stress according to PSS-10 and the length of service of the respondents.

Data analysis did not reveal any statistically significant differences (*p* > 0.05) in the level of burnout according to MBI depending on the workplace. However, length of service correlates significantly (*p* < 0.05) and positively (*r* = 0.486) with emotional exhaustion and (0 = 0.42) depersonalisation; the longer the length of service, the greater the emotional exhaustion and the higher the level of depersonalisation. Length of service correlates significantly (*p* < 0.05) and negatively (*r* = −0.442) with a sense of lack of achievement, i.e., the longer the length of service, the greater the sense of lack of achievement (Table 3).

**Table 3:** Relationship between burnout according to MBI and length of service in the profession, fatigue according to FAS, sleep disorders according to AIS, perceived stress according to PSS-10 and.

Variable	MBI					
	Emotional exhaustion		Depersonalisation		Sense of lack of accomplishment	
	*r*	*p*	*r*	*p*	*r*	*p*
Length of service in the profession [years]	0.486	<0.001	0.43	<0.001	−0.442	<0.001
FAS [points]	0.746	<0.001	0.614	<0.001	−0.713	<0.001
AIS [points]	0.715	<0.001	0.598	<0.001	−0.629	<0.001
PSS-10 [points]	0.498	<0.001	0.389	<0.001	−0.53	<0.001

PPS-10 the perceived stress scale, FAS, fatigue assessment scale, AIS, Athens insomnia scale, MBI, Maslach Burnout Inventory.

### 3.5 Relationships between burnout according to MBI and fatigue according to FAS, sleep disorders according to AIS and the stress level of the respondents according to PSS-10

The level of fatigue according to FAS correlates significantly (*p* < 0.05) and positively (*r* > 0) with emotional exhaustion (*r* = 0.746) and depersonalisation (*r* = 0.614), so the greater the fatigue, the greater the emotional exhaustion and the higher the level of depersonalisation. The level of fatigue according to FAS correlates significantly (*p* < 0.05) and negatively (*r* = −0.713) with a sense of lack of achievement, so the greater the fatigue, the greater the sense of lack of achievement (lower number of points). Insomnia according to AIS correlates significantly (*p* < 0.05) and positively (*r* > 0) with emotional exhaustion (*r* = 0.715) and depersonalisation (*r* = 0.598), so the more severe the insomnia, the greater the emotional exhaustion and the higher the level of depersonalisation. The severity of insomnia according to the AIS correlates significantly (*p* < 0.05) and negatively (r = −0.629) with a sense of lack of achievement, so the more severe the insomnia, the greater the sense of lack of achievement (lower score). The level of stress according to the PSS-10 correlates significantly (*p* < 0.05) and positively (*r* > 0) with emotional exhaustion (*r* = 0.498) and depersonalisation (*r* = 0.389), so the higher the level of stress, the greater the emotional exhaustion and the higher the level of depersonalisation. The stress level according to PSS-10 correlates significantly (*p* < 0.05) and negatively (*r* = −0.53) with the feeling of lack of achievement, so the higher the stress level, the greater the feeling of lack of achievement (lower number of points) (Table 3).

## 4 Discussion

The results of this study confirm the high burden of psychosocial risks among nurses working in shift systems. The findings are consistent with previous research indicating that shift work and high occupational demands contribute to deterioration in nurses’ mental and physical health (34–36). However, unlike many earlier studies that examined single outcomes in isolation, the present study provides an integrated analysis of stress, fatigue, sleep quality, and burnout within one professional group and organizational context.

Although many associations between stress, fatigue, sleep disturbances, and burnout among nurses have been reported previously, the present study adds value by integrating these psychosocial risks within a single analytical framework and organizational context. Rather than treating fatigue as an isolated outcome, the findings support its role as a central indicator within a broader cluster of psychological health risks associated with shift work.

Importantly, the results suggest that fatigue may serve as an early warning signal reflecting cumulative psychosocial strain, closely linked to perceived stress, sleep quality, and burnout dimensions. From an applied perspective, this integrated approach may support the development of screening or alert-based strategies aimed at identifying nurses at increased risk and implementing timely preventive or organizational interventions. While the cross-sectional design does not allow for formal staging or predictive modeling, the present findings provide empirical groundwork for future longitudinal studies focused on fatigue-based risk stratification and intervention planning.

A substantial proportion of the respondents reported high stress levels, moderate to severe fatigue, sleep disturbances, and symptoms of burnout, particularly in the dimension of reduced personal accomplishment. The findings of our present study are consistent with those obtained by Ozga et al. (37), among others, who emphasise that working in conditions of high responsibility and low predictability increases the risk of chronic stress in nurses. Importantly, the level of stress correlated significantly with age and marital status, which may indicate the accumulation of psychosocial burdens in the long term and limited social resources among single or widowed individuals.

More than half of the nurses surveyed reported moderate fatigue, and more than 12% reported severe fatigue. These results confirm the findings of Nowrouzi et al. (38), who showed that nurses’ occupational fatigue is directly related to the number of hours worked, lack of rest and the rotating shift system. In turn, research by Zhang et al. (61)indicates that fatigue is also associated with clinical errors and reduced quality of care (39), which exacerbates health and organisational consequences.

It is worth noting that more than two-thirds of the respondents met the criteria for insomnia, which is alarming given the role of sleep in regulating mental functioning and the body’s immunity. This phenomenon has been extensively described by Radosz (40), who point out that night and shift work seriously disrupts circadian rhythms, leading to sleep disorders and thus an increased risk of depression and cardiovascular disease. In our study, higher insomnia scores were observed among nurses working in the basic system compared with those working rotating shifts. This finding should be interpreted with caution and may reflect difficulties in adapting to fixed early-morning schedules among nurses with longer professional experience rather than contradicting the well-established association between shift work and sleep disturbances reported in the literature. A systematic review and meta-analysis showed that sleep disorders in nurses working night shifts correlate with an increased risk of depression (OR ≈ 1.49) (41, 42). Other studies confirm that shift work, especially night work, significantly increases the risk of mood disorders, including depression (41).

One of the key findings of the study is the significant increase in burnout symptoms among nurses, particularly in terms of a sense of lack of achievement, emotional exhaustion and depersonalisation. These data are consistent with the findings of Maslach and Leiter (62), who indicate that nursing is one of the professions most vulnerable to burnout syndrome, particularly in the context of limited organisational support, lack of recognition and task overload (43). The results of a meta-analysis of other studies showed a significant relationship between burnout and sleep disorders in nurses (44). Another study emphasises that people with shift work disorder are at a significantly higher risk of mental health problems and burnout (45, 46).

The observed relationship between age, education, length of service and the severity of fatigue, insomnia and burnout indicates the importance of personal and professional factors in modulating responses to psychosocial stressors. Similar conclusions can be drawn from the study by Wang et al. (63), where older age and longer seniority were risk factors for emotional exhaustion and depersonalisation (47). Preliminary studies indicate that quick return schedules, i.e., short intervals between shifts, reduce sleep quality and increase the risk of burnout (48). In addition, a study by Al-hrinat et al. (49) showed that stress associated with night shifts indirectly worsens the quality of life of nurses, with sleep disorders acting as a mediator of this relationship. A meta-analysis published in 2024 and other studies have shown that burnout among nurses is associated with reduced quality of care, patient safety and patient satisfaction (50–52).

From an organisational practice perspective, it seems necessary to implement psychoeducational programmes and preventive interventions, including: training in sleep hygiene, flexible work schedules that take into account staff preferences, stress reduction programmes (e.g., based on mindfulness – MBSR) and support and supervision groups. Research confirms that such measures improve nurses’ well-being and reduce symptoms of burnout (53, 54).

Based on the observed distribution of fatigue levels and their consistent associations with perceived stress, sleep disturbances, and burnout dimensions, a pragmatic fatigue staging framework may be proposed for use in hospital settings. Using established Fatigue Assessment Scale (FAS) cut-off values, nurses may be categorized into low, moderate, and high fatigue stages, which can serve as an initial screening and risk-triage tool rather than a diagnostic or predictive model. Importantly, higher fatigue stages were accompanied by a cumulative psychosocial burden, including greater emotional exhaustion, depersonalization, and impaired sleep quality, suggesting that fatigue may function as an early and easily identifiable alert indicator of broader psychological risk (55, 56).

From an organizational perspective, such a staging approach could support proportionate, stage-specific countermeasures. Low fatigue levels may warrant routine monitoring and preventive education, whereas moderate fatigue could prompt schedule review, sleep hygiene interventions, and stress management support. High fatigue levels may signal the need for timely occupational health referral, targeted assessment of sleep disorders, and temporary workload or shift modifications. Although this framework requires validation in longitudinal and multicenter studies, it offers a feasible and low-cost strategy for integrating psychosocial risk monitoring into everyday nursing management and occupational health practice (57, 58).

This study contributes to the existing literature by providing an integrated assessment of stress, fatigue, sleep quality, and burnout among shift-working nurses within a single organizational context. By demonstrating the interrelated nature of these psychosocial risks and identifying sociodemographic and work-related factors associated with their severity, the findings extend current knowledge beyond studies examining single outcomes in isolation (59, 60).

## 5 Limitations and strengths of the study

The study conducted among nursing staff working in shifts provided valuable data on the prevalence of psychosocial health risks such as fatigue, stress, sleep disorders and burnout. However, there are some limitations that may affect the interpretation of the results.

First, the relatively small sample size may limit the statistical power of the analyses and restrict the generalizability of the findings. In addition, the study was conducted in a single clinical facility using a convenience sampling strategy, which may have introduced selection bias. Although the response rate was high (83%), convenience sampling does not ensure full representativeness of the broader nursing population. Nurses working in other healthcare institutions or regions may differ with respect to organizational structure, workload, staffing levels, and workplace culture. Therefore, the results should be interpreted with caution and primarily within the context of similar healthcare settings.

Second, the cross-sectional design of the study, based on a single measurement point, does not allow for assessment of changes over time or for drawing causal inferences between the analyzed variables. Longitudinal studies would be necessary to better understand the directionality and dynamics of relationships between stress, fatigue, sleep disturbances, and burnout.

The use of the self-description technique, although commonly used in psychosocial research, carries the risk of distortions resulting from the subjective perceptions of the respondents, their current mental state and their desire to present themselves in a socially acceptable manner. It is also important to note that the study did not take into account other potentially relevant contextual variables, such as family situation, level of social support, or number of patients per nurse, which could influence the level of perceived stress and fatigue.

Despite these limitations, the study also has significant strengths. First and foremost, it is important to highlight the use of reliable and standardised research tools – the Perceived Stress Scale PSS-10, the Fatigue Assessment Scale FAS, the Athens Insomnia Scale AIS and the MBI Burnout Questionnaire. This enabled a comprehensive and reliable assessment of the areas studied.

Another advantage of the study was its high responsiveness – as many as 83% of the distributed questionnaires were completed and qualified for analysis, which demonstrates the high level of commitment of the participants and strengthens the representativeness of the results. Another unquestionable advantage of the study was the detailed analysis of the socio-demographic determinants of the analysed phenomena, which made it possible to identify the most vulnerable professional groups. The use of appropriate statistical methods, including non-parametric tests and correlation analysis, allowed for the detection of statistically significant relationships and provided a solid basis for formulating practical conclusions.

## 6 Conclusion

- Shift work among nurses is associated with an increased burden of psychosocial health risks, including fatigue, perceived stress, sleep disturbances, and burnout symptoms.
- Age, education and marital status influence the level of fatigue, insomnia, stress and burnout. Older age and lower education in the study group were associated with a greater severity of these phenomena. Higher levels of fatigue, insomnia, perceived stress, and burnout symptoms were observed among widowed and divorced respondents compared with other marital status groups.
- Among the respondents, longer service was associated with higher levels of fatigue, sleep disturbances and emotional exhaustion, as well as greater depersonalisation and a lower sense of professional achievement. The findings indicate that longer professional experience is associated with higher levels of fatigue, sleep disturbances, and burnout symptoms. In contrast, the specific workplace or ward type did not significantly influence these outcomes.
- Higher levels of fatigue, increased insomnia and greater stress among the study group contribute to increased emotional exhaustion and depersonalisation, while at the same time reducing the sense of professional achievement. The results obtained emphasise the need to introduce organizational and preventive strategies aimed at managing fatigue, improving sleep quality, and reducing occupational stress among nurses.
- The high levels of fatigue, stress, sleep disorders and burnout require not only organisational changes, but also the development of a work culture based on support, understanding and care for the well-being of healthcare workers. The findings of the study should form the basis for the development of mental health prevention strategies among the nurses surveyed, including education on sleep hygiene, psychological support, flexible work schedules, stress reduction programmes and anti-burnout measures.

### 6.1 Relevance for clinical practice

The practical dimension of the study is also noteworthy – its results can be a useful source of knowledge for managers and nursing care organisers when designing programmes to support the mental health of healthcare workers. Thus, the study not only enriches the scientific literature in the field of healthcare workers’ health, but may also contribute to improving working conditions and the quality of patient care.

## Data Availability

The original contributions presented in the study are included in the article/supplementary material, further inquiries can be directed to the corresponding author.
